# Evaluation of Three New Strategies to Fight Obesity in Families

**DOI:** 10.1155/2010/751905

**Published:** 2010-09-20

**Authors:** C. Luley, A. Blaik, S. Aronica, J. Dierkes, S. Kropf, S. Westphal

**Affiliations:** ^1^Institute of Clinical Chemistry, Otto von Guericke University, 39120 Magdeburg, Germany; ^2^Institute of Biometry and Medical Informatics, Otto von Guericke University, 39120 Magdeburg, Germany

## Abstract

*Aims*. To evaluate 3 strategies to reduce weight in obese families. *Research design and methods*. 142 obese parents and 119 obese children kept a fat-calorie restriction diet. On top of this diet, the families were randomized in a three-factorial design to one or more of three weight-loss strategies: (1) an additional diet preferring carbohydrates having a low glycemic index (dual diet), (2) financial incentive, and (3) telemonitoring of weight and physical activity. *Results*. All children improved their BMI-SDS by 0.18 ± 0.25 (*P* < .001) independently of the weight-loss strategy. In parents, relative losses of weight (kg) were −6.4% versus −4.0% for dual diet versus calorie restriction (*P* = .029), −6.9% versus −3.4% for with or without financial incentive (*P* = .002), and −8.0% versus −4.8% for with or without telemonitoring (*P* = .033). The weight loss after financial incentive plus dual diet plus telemonitoring was −14.4%. *Conclusions*. All strategies were effective in adults, in particular when combined. Children improved their BMI-SDS regardless of the strategy.

## 1. Introduction

The roots of common obesity lie in an imbalance between energy intake with food and energy expenditure due to physical activity. Although an improvement of both is strongly advocated by the medical profession, the media, and even politicians, the successes in weight reduction are unsatisfactory, because obesity continues to spread in affluent societies [1]. Epidemiologists estimate that the growing rates of morbidity and mortality associated with obesity might even reduce the life expectancy of the US population in the 21st century [2].

A basic and widely used approach to weight reduction is calorie restriction. However, experience shows that—in free-living populations—adherence to this measure is difficult, that the resulting weight losses are often suboptimal, and that going off the diet is frequently followed by a rebound weight gain [3]. The second root of obesity, insufficient physical activity, is even more difficult to influence in societies accustomed to motorized transport and remote controls at home.

We, therefore, proposed to evaluate the effects of alternative weight-reduction strategies, implemented on top of a fat calorie-restriction diet [4]. The target group of our study comprised not single obese individuals but obese families consisting of at least one obese parent and at least one obese child. A primary intention behind this selection was to include children, in whom overweight is particularly difficult to curb and in whom long-term consequences are even more severe than in adults. 

The family approach reflects that childhood obesity in primary school-aged children cannot be solved by themselves as they are heavily dependent on their parents [5]. However, weight reduction in families as a whole would be difficult to evaluate, and therefore the effect of weight reduction was evaluated separately for adults and children. 

 All study participants were equally advised to follow a fat-calorie restriction diet and to reduce their daily calorie input by 500 kcal. On top of this basic measure, three additional strategies for weight loss were added and compared in this randomized study following a three-factorial design.

The first strategy concerned nutrition and consisted—in addition to the basic fat-calorie restriction—of the preference of carbohydrate which hardly raise insulin (“dual diet”). The second strategy was a financial incentive, with payments for each kilogram of weight loss in the parents and for each reduction in body mass index standard deviation score (BMI-SDS) in the children. The third strategy was the use of telemetric devices consisting of weighing scales and accelerometers issued to the participants. The data of both were transmitted regularly and enabled us to respond with weekly letters for information and motivation. For each of these weight-reduction strategies, there was a control group treated in the same way except for the additional strategy.

## 2. Subjects and Methods

### 2.1. Selection of Participants and Study Design

The participating families were recruited by means of newspaper advertisements in the area around the German city of Magdeburg. The children had to be older than 7 years to ensure that they were able to read, and younger than 13 to minimize interferences due to puberty. 177 families responded by telephone and received a letter describing the aim and character of the study. 110 families then decided to participate and were invited to the first of four meetings with intervals of one week between successive meetings. At the first meeting, we informed the participants about the project, explained the dietary questionnaires, and randomized them by lot to the various weight-reduction strategies. At the second meeting, anthropometric data were collected, together with dietary questionnaires. Overweight in adults was defined on the basis of their BMI (weight in kilograms divided by height in meters squared) according to the WHO definition [6]. Because the BMI of children is age- and sex-dependent, the respective definitions in children were done in comparison to a German reference population in the year 2001 according to Kromeyer-Hauschild [7]: overweight was defined as a BMI between the 90th and the 97th percentile, and obesity as a BMI exceeding the 97th percentile [8].

 During the third meeting, all participants were informed about the energy metabolism of the human body, energy contents of a representative variety of foodstuffs, and energy expenditure by means of physical activity. At the end of the meeting, participants were randomized by lot to one of the treatment options, but they were not blinded to the different study treatments. The lottery was made by nonscientific personnel of the institute that was not involved into the study. However, all participants were aware of the financial incentive and the telemonitoring options. To ensure adherence, the information on the different dietary treatment options was given in a very general way, and detailed information was only given to those participants that were randomized to the particular dietary treatment. 

In the calorie-restriction group, further information was given using various practical examples. In the group on the combination diet (dual diet) information was given about carbohydrate metabolism and about the glycemic index of carbohydrates, again using many practical examples. Thereafter, the families were no longer contacted until the control day 6 months later. An exception was the group using the telemonitoring devices, in which each participant received a weekly letter. 

**Figure 1:**
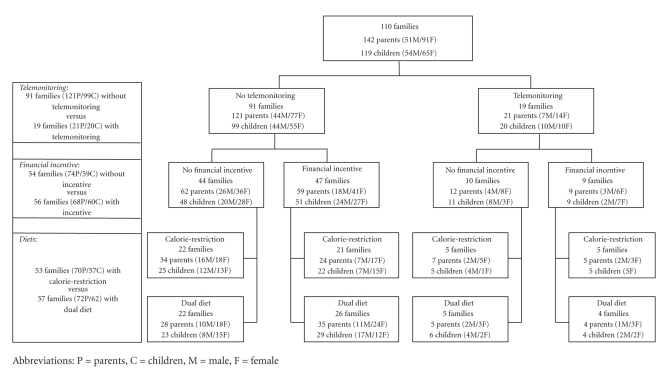
Distribution of families between the additional weight-reduction strategies. The design permits a comparison of the three strategies and also of different combinations of these strategies.

The study was conducted in different waves, since the department must be able to handle so many participants. We calculated beforehand that we would be able to handle about 35 families at one time and planned to have 3 waves of study each lasting 6 months. In each wave, each treatment was offered and every participant had the opportunity to be randomized to one of the treatments with the exception that the telemonitoring was only offered during the 2nd and the 3rd of three waves, but not in the first wave. The parallel study followed a three-factorial design as illustrated in Figure 1. The diagram shows the numbers of families randomized to each weight-reduction strategy including the numbers of parents and children and the sex distribution in each group. The boxes on the left show the numbers of families used for the evaluation of the three weight-loss strategies: telemonitoring, financial incentive, and dual diet. It should be remembered that the groups shown on the left in Figure 1 are homogeneous for the weight-loss strategy to be tested (either presence or absence) but heterogeneous for the other strategies evaluated in this study. In the financial incentive group, for example, some are also on the dual diet and/or telemonitoring while others are not. For the comparison financial incentive versus no financial incentive, however, the distribution of additional strategies is equal in both groups so that the effects of these additional strategies are counterbalanced. The design, therefore, also allows an evaluation of strategy combinations, although in smaller group sizes, in particular in the branch using the telemetry equipment. The distribution into these different treatment groups is given for the parents and the children in the top part of Tables 1(a) and 1(b).

### 2.2. Interventions

#### 2.2.1. Telemonitoring

The telemedical equipment consisted of a weighing scale for each family, an accelerometer for each participant, and a Homebox for each family which received the data from the scale and the accelerometers via Bluetooth and transferred them via a telephone link to a server in Munich. All instruments were bought from Aipermon GmbH, Munich, Germany. The data were transferred to a server in Magdeburg university hospital where weekly reports were generated and sent to each participant of the telemonitoring group. Each report gave the individual's weight curve from the beginning of the project and a graph showing for each day of the past week the duration of activity as a percentage of 24 hours, bars with four colours representing four different activity levels from active to sporty, the distance covered in kilometres, and the motoric kcal burned. Each letter also contained comments assessing progress over the past week and aiming to motivate the participant. 

#### 2.2.2. Financial Incentive

For the parents, the financial incentive was 5 Euros for every kilogram of weight loss. For children the weight loss was calculated differently, taking into account the individual need of each child to lose weight. Children with a body mass index between the 90th and 97th age-adjusted BMI-percentile were asked to maintain their weight and were paid in dependence on how well they managed to achieve this goal. Children with age-adjusted BMI-percentiles between 97th and 99th, or above the 99th age-adjusted BMI-percentile received 5 Euros per weight losses of, respectively, 500 g or 1 kg.

#### 2.2.3. Diets

All participants received a conventional low-fat diet according to recommendations issued by the Deutsche Gesellschaft für Ernährung [4]. The target macronutrient composition was more than 55% energy from carbohydrates, less than 30% from fat, and 15% energy from protein. The basic diet for all participants was supported by a list giving the calorie contents of a large variety of foodstuffs [9]. The dual diet group kept this basic diet but was additionally asked to replace high GI carbohydrates with low GI carbohydrates. Emphasis was placed on the fact that carbohydrates should only be replaced but not avoided as required by the Atkins diet [10]. Special attention was paid to the amount of sugar in the diet. Composition of monosaccharides and disaccharides were explained (in simple wording) to the participants, to enable them to avoid foods with high content of mono- and disaccharides. The dual diet group received a second list giving the glycemic index (GI) and the content of glucose and sucrose for a large variety of carbohydrates [11]. 

All participants were advised to reduce their daily energy intake by at least 500 kcal. Dietary changes were monitored by means of 3-day food records. These records were completed for the first time before the first dietary training and then again one week before the control visit at 6 months. The data in these records were evaluated by a computerized programme (DGE-PC, Deutsche Gesellschaft für Ernährung, Bonn, version II.2). The program is based on the Bundeslebensmittelschlüssel II.2 which contains the macronutrient and micronutrient content of >10000 foodstuffs commonly consumed in Germany [12]. The proportion of carbohydrates with refined carbohydrates was assessed by adding the sucrose and glucose contents and expressing this sum as a percentage of total carbohydrates.

### 2.3. Statistical Methods

The statistical analysis of the data was done using the SAS package, version 9.1 [13]. Adults and children were considered separately. We considered weight loss as primary endpoint. It was expressed as relative weight loss (%) in the adults and a change of the BMI-SDS in the children. Changes in nutrient intakes were considered secondary endpoints. 

Regarding the problem of missing second visits, analyses were performed both on the basis of the last observation carried forward (LOCF) procedure and with the available data. The LOCF procedure was also used for the children, with the exception of BMI and the body weight. In this case, because children continued to grow during the 6 months of the study, a mean growth of 3 cm in height was assumed. The body weight after 6 months was also assessed with the assumption that the children remained on the same weight percentile as at the start of the study. Because of the small proportion of families with more than one adult or more than one child, possible dependencies between members of the same family were ignored.

In a first step, the target variable was analysed by three-factorial ANOVA (PROC GLM) with the factors telemonitoring, financial incentive, and dual diet including all pairwise interaction terms. As it turned out, the interaction terms were not significant; confidence intervals for the three effects were estimated from a model with the main effects only. In order to distinguish more clearly between the effects of different combinations of the factors, a one-factorial ANOVA was then carried out with the one-sided Dunnett test as a post hoc comparison, using the maximally supported group (telemonitoring plus financial incentive plus dual diet) as comparator for the other groups. In order to obtain a higher power, a step-down version of this test [14] was used, which controls the type I familywise error rate in a strong sense. We realized this procedure with the function PROBMC in SAS to recalculate the *P*-values from the original Dunnett test.

In the analyses of the children, the step-down steps did not come into effect because of missing significant results in the primary step. Here, an additional paired t-test was applied for the total group (disregarding different strategies) to check the overall effect of the programme.

## 3. Results

### 3.1. Characteristics of the Participants

Tables 1(a) and 1(b) show the numbers of cases and the physical and biochemical characteristics of the parents and children grouped according to their specific combination of weight-loss strategies. As required by the inclusion criteria, both the parents and the children were obese. Parents had a mean BMI of 33 kg/m^2^ and children had a mean elevated BMI standard deviation score of 2.03. The parents were relatively young with a mean age of 39 years and were clinically healthy. However, pathological biochemistry was observed in the parents for glucose (>6.4 mmol/l) in 5% of the cases, for insulin (>100 pmol/l) in 10%, for systolic and/or diastolic blood pressure (>130/80 mm Hg) in 36%, for triglycerides (>2.3 mmol/l) in 14%, for LDL-cholesterol (>4.0 mmol/l) in 24% and for in HDL-cholesterol (♀ < 1.17 and ♂ < 0.91 mmol/l) in 36%.

### 3.2. Dropout Rates

**Figure 2:**
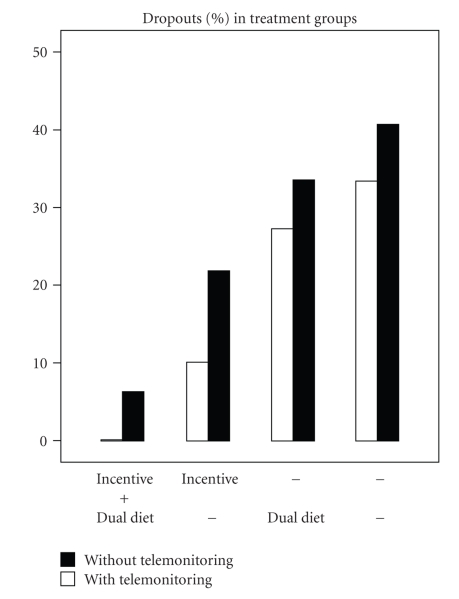
Dropout rates in groups with different combination of weight-reduction strategies. Parents and children evaluated together.

Figure 2 shows the dropout rates for both parents and children together. Children dropped out more often (12%) than their parents (8%, *P* = .015), independently of the weight-reduction strategy. The dropout rate showed extremes for the comparison groups with and without financial incentive: with the incentive it was lowest (at 9%) in the parents and highest (at 39%) in the children without the financial incentive (*P* = .001). Also, the other two strategies—dual diet and telemonitoring—had lower dropout rates than the groups without each strategy, although not statistically significant.

### 3.3. Weight Loss and Dietary Intake

The weight reductions and the changes in nutrient intake after the implementation of the three additional strategies are given for the parents in Tables 2(a)–2(c) and for the children in Tables 3(a)–3(c). The tables show on the left the results from the participants appearing after 6 months (“completers”) and on the right the results calculated on the basis of the last observation carried forward (LOCF), which also includes the dropouts. The completers group is identical with those patients who complied with the study protocol, and the LOCF group equals an analysis according to “intention to treat”. 

Each additional strategy proved to be effective in the parents. The additional relative weight loss in the “completeters” with 95% confidence interval (estimated from the ANOVA model, thus corrected for influences of the other factors) was 2.2% (0.2%, 4.3%) in the dual diet group, 3.3% (1.2%, 5.3%) in the financial incentive group, and 3.7% (0.9%, 6.5%) in the telemonitoring group.

The children also derived benefit from participation in this study. Although in absolute terms they gained between 1 and 2 kg weight, their standard deviation score (SDS)—taking into account the weight gain due to body growth in the course of the study—decreased. This decrease between baseline and 6 months was 0.18 ± 0.25 (*P* < .001) for all children and ranged from 0.16 to 0.21 for the three strategy comparisons. Differences between strategies, however, were not statistically significant (Table 3). The financial incentive may possibly have had an effect, but this was statistically significant only in the LOCF evaluation, which might have overestimated the difference. 

As for the interaction within families, there was no correlation found between the weight losses in parents on the one hand and the weight losses in their children on the other hand. However, in the 28% of all families in which two parents participated, there was a close correlation between the weight losses of the two adults (*r* = 0.767, *P* < .000).

Tables 2 and 3 also show changes in macronutrient intake. A limitation of the dietary assessment is that this was only measured at the beginning and the end of the study period but not in between. Nevertheless, almost all nutrients were reported to be consumed less by all groups at the control visit after the 6 months. Some of the strategies tested were associated with statistically different macronutrient intakes: the parents on the dual diet ate less fat and took in fewer calories, but their consumption of refined carbohydrates showed only borderline difference from that in the calorie-restriction alone group (Table 2(a)). Less carbohydrate was consumed by the parents with a financial incentive (Table 2(b)). Surprisingly, the proportion of glycemic carbohydrates actually increased in parents in the telemonitoring group (Table 2(c)) in contrast to their children in whom the consumption of refined carbohydrates was lower (Table 3(c)). Other statistical significant changes in nutrient intake were not found in children with the exception of a lower consumption of calories in the dual diet group (Table 3(a), LOCF evaluation)—like in their parents (Table 2(a)). 

**Figure 3:**
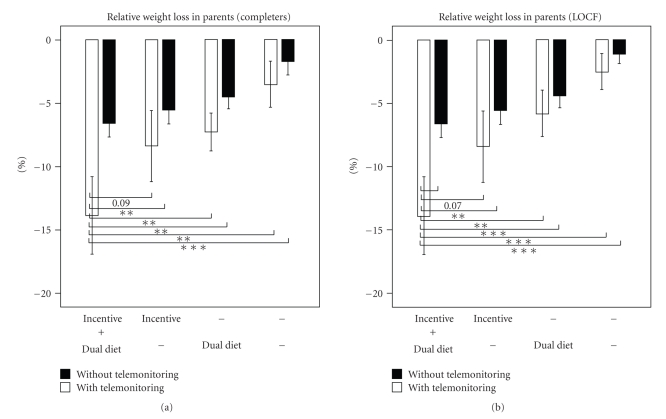
Weight loss in groups of parents with different combinations of weight-reduction strategies. Statistically significant differences are indicated by *(*P* < .05), **(*P* < .01), and ***(*P* < .001).

Combination of the additional strategies markedly improved the dropout rates and the losses of weight. Figure 2 shows the dropout rates in the parents and children taken together and their wide range of variation from 40% in individuals on calorie restriction alone down to 0% in the group on the combination of telemonitoring plus financial incentive plus dual diet. The weight losses in parents from different strategy groups are shown in Figure 3 (completers). The weight loss once again increases with increasing number of additional strategies, from 2.8% in parents on calorie restriction up to 14.4% in those receiving the “full program”. A similar trend was observed for BMI-SDS in the children, although to a smaller extent and without reaching statistically significance (data not shown).

## 4. Discussion

In this randomized and controlled trial, we evaluated three additional strategies aimed at reducing weight in obese families. The major results are as follows: (1) in parents, all three strategies were effective; (2) in children, improvement of BMI-SDS was independent of the strategies; (3) combinations of these new strategies enhance compliance and loss of weight in parents.

The better effect of the dual diet compared with calorie restriction alone could not readily be foreseen. Studies of the effects of carbohydrate diets with different glycemic index have given contradictory results in the past particularly in respect to weight loss [15]. However, the majority of recent studies indicate that low-GI carbohydrates curb appetite [16], reduce the energy intake in subsequent meals [17], and improve health markers [18, 19]. Our study differs from most of these studies in that it did not investigate the impact of a low-GI diet alone but the effect of a low-GI diet on top of calorie restriction. The dietary records show that the better weight loss in the dual diet group can be explained by consumption of fewer calories (Tables 2(a) and 3(a)) due to the smaller intake of fat. This may have been caused by smaller appetite and consequently a smaller energy intake, as mentioned above. 

It might well have been expected that financial incentive would improve both weight loss and compliance. However, the implications of this observation remain to be debated. Further studies should investigate if this improvement is lasting and if the health benefit justifies the costs. Its effectiveness in adults could be of interest for health insurance companies, which might offer bonuses to obese individuals to encourage them to lose weight and consequently to improve risk markers. Further studies should determine the magnitudes and frequencies of such bonuses necessary to motivate weight losses. 

Telemetric monitoring of weight and physical activity is a new and effective strategy. Two separate mechanisms are involved here. The first acts via the continuous feed back from the accelerometer telling the user how effective physical activity has been in terms of the distances covered and—more importantly—in terms of the calories used up. We noticed that the users pay considerable attention to this information—they try to increase their daily activity and to maintain it at a high level. This instrument therefore works like a personal coach, who steadily brings to the user's awareness his or her physical activity and the associated benefit. The second mechanism is the regular feedback from the person in charge. The families using these devices were asked to use the scales every day. They reported unanimously that the knowledge that their body weight was continually under observation provided an additional stimulus to control food intake and to increase activity during the day. They also reported that they eagerly awaited the weekly letters, which commented on their progress and encouraged them to continue. Taken together, these strategies proved to be an effective tool enhancing their physical activity and boosting their motivation. This technology does both without frequent and time-consuming meetings and also over long distances. It must also be taken into account that telemonitoring is comprised of several elements all of which can contribute to a better weight reduction: daily self-weighing, [20–22] increase of physical activity, and regular feedback for motivation. 

The children who completed the study lowered their BMI-SDS by 0.16 to 0.21 largely independent of the strategies. Because BMI-SDS is an overweight measure which is not easily understood, we illustrate the BMI-SDS change in a hypothetical child: a 10 years old girl with a body weight of 59 kg and 150 cm height would be on the 99th BMI percentile of her age group and her BMI-SDS would be 2.32. Six months later she would have grown by 3 cm. Assuming that her BMI-SDS would be unchanged, her weight gain due to body growth would be 3.6 kg. A reduction of her BMI-SDS by 0.2 (as in this study) would lower this weight gain to 0.5 kg. Therefore, this girl would have avoided an increase of weight by 3.1 kg which has about the same order of magnitude of weight loss as in their parents, namely by 5.1%.

In parents, the combinations of the strategies were more effective than each strategy alone. This applied both to the dropout rates (Figure 2) and to the losses of weight (Figure 3). The combination of telemonitoring plus financial incentive plus dual diet proved to be most effective. In the group of parents taking advantage of all three strategies there were no dropouts and the mean weight loss was 14.4%. However, it must be taken into account that the group sizes for the comparisons of combined strategies were lower than for the comparisons of single strategies (Figure 1 and Table 1). This was particularly the case when telemonitoring was involved which led to group sizes between *n* = 4 and *n* = 7. Although the figures suggest a marked superiority of strategy combinations, this finding remains to be confirmed with larger sample sizes. 

Are weight losses after these alternative strategies more sustained than those after calorie restriction? While the data cannot answer this question, we are optimistic that the accelerometers open a new road to their users by improving their awareness of everyday physical activity. Progress in weight control by increased physical activity has been difficult in the past, but may become easier with the use of the telemonitoring. In fact, the dual diet and the enhancement of physical activity are key elements of the desired change in lifestyle, and they are continually reinforced by telemetric control, a kind of lifestyle training. 

Summing up, this study shows that in adults weight reduction by fat-calorie restriction can be improved by three additional strategies. Combining these strategies enhances the weight loss remarkably. In children, however, the collective family endeavour seems to be more important than the chosen strategy.

## Figures and Tables

**Table 1a:** (a) Baseline characteristics in adults (means ± standard deviations). “+” measure present, “−” measure absent.

Telemedicine	−	−	−	−	+	+	+	+	Total
Financial benefit	−	−	+	+	−	−	+	+	
Dual diet	−	+	−	+	−	+	−	+	
Calorie restriction	+	+	+	+	+	+	+	+	
*N*	34	28	24	35	7	5	5	4	142
Age (*y*)	41 ± 6	38 ± 4	40 ± 7	38 ± 4	40 ± 8	39 ± 5	39 ± 4	38 ± 5	39 ± 5
Male/female (*n*)	16/18	10/18	7/17	11/24	2/5	2/3	2/3	1/3	51/91
Smoking status, *n* (%)									
current	11 (32.4)	10 (35.7)	4 (16.7)	10 (28.6)	/	/	1 (20.0)	/	36 (25.4)
former	12 (35.3)	10 (35.7)	6 (25.0)	14 (40.0)	/	1 (20.0)	1 (20.0)	1 (25.0)	45 (31.7)
never	11 (32.4)	8 (28.6)	14 (58.3)	11 (31.4)	7 (100)	4 (80.0)	3 (60.0)	3 (75.0)	61 (43.0)
Hyperglycemia, n (%)	3 (8.8)	1 (3.6)	1 (4.2)	2 (5.7)	/	/	/	/	7 (4.9)
BMI (kg/m²)	34 ± 6	33 ± 5	33 ± 6	33 ± 5	34 ± 8	33 ± 4	31 ± 3	37 ± 3	33 ± 6
Height (cm)	175 ± 9	172 ± 8	170 ± 9	170 ± 9	171 ± 13	175 ± 7	171 ± 12	168 ± 8	172 ± 9
Weight (kg)	104 ± 19	99 ± 17	95 ± 21	95 ± 16	102 ± 32	100 ± 12	90 ± 12	105 ± 17	99 ± 19
Waist circumference (cm)	110 ± 13	107 ± 12	106 ± 13	106 ± 13	99 ± 8	105 ± 8	102 ± 12	114 ± 12	107 ± 12
Systolic BP (mmHg)	138 ± 20	132 ± 11	130 ± 22	127 ± 23	121 ± 22	132 ± 19	126 ± 15	119 ± 19	131 ± 20
Diastolic BP (mmHg)	87 ± 11	84 ± 17	82 ± 12	82 ± 11	76 ± 14	80 ± 7	82 ± 13	78 ± 15	83 ± 12
Glucose (mmol/L)	5.3 ± 0.6	5.4 ± 1.1	5.4 ± 1.5	5.2 ± 0.6	5.1 ± 0.5	5.5 ± 0.3	5.1 ± 0.3	5.1 ± 0.3	5.3 ± 0.9
Insulin (pmol/L)	62 ± 37	49 ± 23	51 ± 29	57 ± 31	50 ± 23	95 ± 66	54 ± 16	87 ± 37	57 ± 33
Total Cholesterol (mmol/L)	5.9 ± 1.1	5.3 ± 0.9	5.2 ± 1.1	5.1 ± 1.0	5.2 ± 0.8	5.2 ± 1.0	5.0 ± 0.6	4.9 ± 0.8	5.3 ± 1.0
LDL cholesterol (mmol/L)	3.9 ± 1.1	3.6 ± 0.8	3.1 ± 0.9	3.3 ± 0.9	3.5 ± 0.9	3.5 ± 1.0	3.4 ± 0.5	3.2 ± 0.8	3.5 ± 0.9
HDL cholesterol (mmol/L)	1.2 ± 0.3	1.2 ± 0.3	1.4 ± 0.4	1.2 ± 0.3	1.2 ± 0.2	1.2 ± 0.4	1.2 ± 0.2	1.3 ± 0.3	1.2 ± 0.3
Triacylgylcerol (mmol/L)	1.8 ± 1.0	1.3 ± 0.8	1.8 ± 2.1	1.5 ± 1.1	1.1 ± 0.4	1.5 ± 0.8	1.1 ± 0.4	1.3 ± 0.7	1.5 ± 1.2
hs-CRP (mg/L)	5.3 ± 6.8	3.3 ± 3.7	4.2 ± 4.1	4.3 ± 5.2	2.5 ± 1.8	3.6 ± 2.0	21.7 ± 25.1	2.7 ± 1.7	4.8 ± 7.2

**Table 1b:** (b) Baseline characteristics in children (means ± standard deviations). “+” measure present, “−” measure absent.

Telemedicine	−	−	−	−	+	+	+	+	Total
Financial benefit	−	−	+	+	−	−	+	+	
Dual diet	−	+	−	+	−	+	−	+	
Calorie restriction	+	+	+	+	+	+	+	+	
*N*	25	23	22	29	5	6	5	4	119
Age (*y*)	10 ± 2	10 ± 2	10 ± 3	11 ± 3	10 ± 2	10 ± 2	9 ± 2	10 ± 2	10 ± 2
Male/female (*n*)	12/13	8/15	7/15	17/12	4/1	4/2	0/5	2/2	54/65
BMI (kg/m²)	26 ± 5	26 ± 6	24 ± 3	26 ± 4	26 ± 7	25 ± 5	23 ± 4	27 ± 7	25 ± 5
BMI-SDS	2.08 ± 0.63	2.06 ± 0.70	1.94 ± 0.40	2.11 ± 0.49	1.99 ± 0.69	1.98 ± 0.62	1.88 ± 0.54	1.74 ± 0.41	2.03 ± 0.56
Height (cm)	151 ± 14	150 ± 11	148 ± 13	152 ± 14	148 ± 9	153 ± 15	145 ± 9	146 ± 19	150 ± 13
Weight (kg)	60 ± 21	60 ± 21	54 ± 17	61 ± 19	57 ± 20	61 ± 21	50 ± 12	51 ± 21	58 ± 19
Systolic BP (mmHg)	111 ± 16	109 ± 14	111 ± 20	106 ± 17	102 ± 10	97 ± 10	105 ± 19	116 ± 23	108 ± 17
Diastolic BP (mmHg)	70 ± 11	72 ± 11	73 ± 14	66 ± 11	58 ± 8	58 ± 8	55 ± 11	66 ± 5	68 ± 12
Glucose (mmol/L)	4.9 ± 0.3	4.9 ± 0.4	4.9 ± 0.4	4.9 ± 0.4	5.0 ± 0.4	5.0 ± 0.2	4.6 ± 0.5	4.9 ± 0.3	4.9 ± 0.4
Insulin (pmol/L)	56 ± 28	50 ± 26	52 ± 28	56 ± 34	71 ± 75	47 ± 9	64 ± 55	73 ± 66	56 ± 34
Total Cholesterol (mmol/L)	4.5 ± 0.9	4.3 ± 0.7	4.6 ± 1.0	4.3 ± 1.0	3.9 ± 0.8	3.9 ± 0.8	4.0 ± 0.4	4.7 ± 0.3	4.4 ± 0.9
LDL cholesterol (mmol/L)	2.9 ± 0.7	2.6 ± 0.6	2.9 ± 0.8	2.7 ± 0.9	2.5 ± 0.5	2.4 ± 0.6	2.6 ± 0.4	2.7 ± 0.4	2.7 ± 0.7
HDL cholesterol (mmol/L)	1.2 ± 0.2	1.3 ± 0.3	1.3 ± 0.3	1.2 ± 0.2	1.2 ± 0.4	1.2 ± 0.3	1.0 ± 0.1	1.4 ± 0.2	1.2 ± 0.3
Triacylgylcerol (mmol/L)	1.0 ± 0.5	1.0 ± 0.6	1.1 ± 0.7	1.1 ± 0.6	0.8 ± 0.5	1.1 ± 0.8	1.2 ± 0.2	0.8 ± 0.3	1.0 ± 0.6
hs-CRP (mg/L)	2.2 ± 1.8	2.8 ± 5.8	2.4 ± 4.2	2.1 ± 2.0	1.0 ± 0.7	3.5 ± 4.1	6.1 ± 10.5	1.0 ± 0.7	2.4 ± 3.9

**Table 2a:** (a) Impact of diets on weight loss and nutrient intakes in parents (means ± standard deviations).

	Completers			Last observation carried forward		
	Calorie restriction	Dual Diet	*P*	Calorie restriction	Dual Diet	*P*

*N*	50	61		70	72	
Δ-weight (%)	−4.0 ± 5.3	−6.4 ± 6.0	.029	−2.9 ± 4.9	−6.0 ± 6.1	.001

Nutrients						
*N*	41	54		57	64	
Δ-Kcal per day	−305 ± 573	−545 ± 579	.048	−235 ± 526	−482 ± 557	.014
Δ-Fat per day (g)	−10.3 ± 35	−31.3 ± 30	.003	−8.0 ± 31	−27.5 ± 29	.001
Δ-Protein per day (g)	−10.6 ± 27	−14.0 ± 25	n.s.	−7.1 ± 24	−11.0 ± 25	n.s.
Δ-CH per day (g)	−38.3 ± 68	−47.9 ± 68	n.s.	−31.6 ± 61	−44.6 ± 65	n.s.
Δ-RCH per day (%) of CH	+2.3 ± 14	−2.7 ± 12	(.057)	+1.1 ± 12	−2.5 ± 11	(.08)

Abbreviations: CH = Carbohydrates, RCH = refined carbohydrates (sucrose plus glucose).

**Table 2b:** (b) Impact of a financial incentive on weight loss and nutrient intakes in parents (means ± standard deviations).

	Completers			Last observation carried forward		
	Without incentive	With incentive	*P*	Without incentive	With incentive	*P*

*N*	49	62		74	68	

Δ-weight (%)	−3.4 ± 4.6	−6.9 ± 6.2	.002	−2.8 ± 4.7	−6.3 ± 6.2	.000
Nutrients						
*N*	42	53		63	58	
Δ-Kcal per day	−379 ± 527	−491 ± 629	n.s.	−299 ± 470	−438 ± 630	n.s.
Δ-Fat per day (g)	−22.2 ± 27	−22.3 ± 39	n.s.	− 16.9 ± 24	−19.8 ± 38	n.s.
Δ-Protein per day (g)	−13.0 ± 27	−12.2 ± 25	n.s.	− 8.9 ± 23	−9.4 ± 26	n.s.
Δ-CH per day (g)	−27.6 ± 58	−56.3 ± 73	.039	−24.9 ± 53	−53.2 ± 71	.015
Δ-RCH per day (%) of CH	−0.4 ± 14	−0.6 ± 11	n.s.	−0.5 ± 12	−1.1 ± 11	n.s.

Abbreviations: CH = Carbohydrates, RCH = refined carbohydrates (sucrose plus glucose).

**Table 2c:** (c) Impact of telemonitoring on weight loss and nutrient intakes in parents (means ± standard deviations).

	Completers			Last observation carried forward		
	Without telemonitoring	With Telemonitoring	*P*	Without telemonitoring	With Telemonitoring	*P*

*N*	93	18		121	21	
Δ-weight (%)	−4.8 ± 5.7	−8.0 ± 6.0	.033	−4.1 ± 5.6	−6.9 ± 6.2	.041

Nutrients						
*N*	83	12		106	15	
Δ-Kcal per day	−449 ± 599	−389 ± 504	n.s.	−374 ± 566	−311 ± 475	n.s.
Δ-Fat per day (g)	−21.4 ± 35	−27.8 ±24	n.s.	− 17.7 ± 32	−22.3 ± 24	n.s.
Δ-Protein per day (g)	−12.6 ±26	−12.1 ± 28	n.s.	− 9.1 ± 24	−9.6 ± 25	n.s.
Δ-CH per day (g)	−48.6 ± 67	−10.8 ± 67	n.s.	−42.7 ± 63	−8.6 ± 59	n.s.
Δ-RCH per day (%) of CH	−1.9 ± 12	+9.2 ± 11	.004	−2.0 ± 11	+7.3 ± 11	.003

Abbreviations: CH = Carbohydrates, RCH = refined carbohydrates (sucrose plus glucose).

**Table 3a:** (a) Impact of diets on weight loss and nutrient intakes in children (means ± standard deviations).

	Completers			Last observation carried forward		
	Calorie restriction	Dual diet	*P*	Calorie restriction	Dual Diet	*P*
*N*	38	49		57	62	
Δ-weight (%)	2.7 ± 5.5	2.3 ± 4.9	n.s.	2.4 ± 4.7	2.2 ± 4.6	n.s.
Δ-SDS	−0.18 ± 0.3	−0.19 ± 0.2	n.s.	−0.13 ± 0.24	−0.15 ± 0.23	n.s.

Nutrients						
*N*	34	42		49	53	
Δ-Kcal per day	−96 ± 396	−239 ± 475	n.s.	−37 ± 377	−205 ± 438	.041
Δ-Fat per day (g)	−4 ± 55	−11 ± 24	n.s.	−2.0 ± 23	−10 ± 23	(.074)
Δ-Protein per day (g)	−0.3 ± 19	−3 ± 19	n.s.	1.5 ± 18	−4.1 ± 17	n.s.
Δ-CH per day (g)	−16 ± 58	−28 ± 69	n.s.	−6.7 ± 54	−23 ± 63	n.s.
Δ-RCH per day (%) of CH	−2.1 ± 13	−6.2 ± 13	n.s.	−0.9 ± 12	−5.3 ± 12	(.068)

Abbreviations: CH = Carbohydrates, RCH = refined carbohydrates (sucrose plus glucose).

**Table 3b:** (b) Impact of a financial incentive on weight loss and nutrient intakes in children (means ± standard deviations).

	Completers			Last observation carried forward		
	Without incentive	With Incentive	*P*	Without incentive	With Incentive	*P*
*N*	36	51		59	60	
Δ-weight (%)	3.3 ± 5.3	2.0 ± 5.0	n.s.	2.7 ± 4.6	1.9 ± 4.7	n.s.
Δ-SDS	−0.16 ± 0.2	−0.21 ± 0.3	n.s.	−0.09 ± 0.19	− 0.19 ± 0.26	.024

Nutrients						
*N*	31	45		50	52	
Δ-Kcal per day	−167 ± 450	−180 ± 445	n.s.	−115 ± 374	−132 ± 458	n.s.
Δ-Fat per day (g)	−8.4 ± 26	−7.5 ± 24	n.s.	−7.2 ± 23	−5.5 ± 24	n.s.
Δ-Protein per day (g)	−2.9 ± 20	−3.6 ± 18	n.s.	−1.8 ± 16	−1.0 ± 20	n.s.
Δ-CH per day (g)	−20 ± 68	−24 ± 62	n.s.	−11 ± 56	−19 ± 63	n.s.
Δ-RCH per day (%) of CH	−4.5 ± 14	−4.2 ± 13	n.s.	−3.2 ± 11	−3.2 ± 12	n.s.

Abbreviations: CH = Carbohydrates, RCH = refined carbohydrates (sucrose plus glucose).

**Table 3c:** (c) Impact of telemonitoring on weight loss and nutrient intakes in children (means ± standard deviations).

	Completers			Last observation carried forward		
	Without telemonitoring	With telemonitoring	*P*	Without telemonitoring	With telemonitoring	*P*
*N*	72	15		99	20	
Δ-weight (%)	2.5 ± 5.3	2.5 ± 4.6	n.s.	2.4 ± 4.8	1.9 ± 4.1	n.s.
Δ-SDS	−0.18 ± 0.25	−0.20 ± 0.26	n.s.	−0.14 ± 0.23	−0.15 ± 0.24	n.s.

Nutrients						
*N*	65	11		86	16	
Δ-Kcal per day	−154 ± 449	−296 ± 417	n.s.	−109 ± 425	−203 ± 369	n.s.
Δ-Fat per day (g)	−6.6 ± 24	−16 ± 30	n.s.	−5.5 ± 23	−10.8 ± 26	n.s.
Δ-Protein per day (g)	−2.2 ± 19	−10 ± 20	n.s.	−0.4 ± 18	−6.8 ± 17	n.s.
Δ-CH per day (g)	−21 ± 67	−29 ± 47	n.s.	−14 ± 63	−20 ± 41	n.s.
Δ-RCH per day (%) of CH	−3.1 ± 13	−11.8 ± 14	.043	−2.3 ± 12	−8.1 ± 13	(.075)

Abbreviations: CH = Carbohydrates, RCH = refined carbohydrates (sucrose plus glucose).
